# Animal Welfare Assessment and Meat Quality through Assessment of Stress Biomarkers in Fattening Pigs with and without Visible Damage during Slaughter

**DOI:** 10.3390/ani14050700

**Published:** 2024-02-23

**Authors:** Natália Nami Ogawa, Giovanna Lima Silva, Ana Paula Ayub da Costa Barbon, Karina Keller Marques da Costa Flaiban, Caio Abercio da Silva, Luiene Moura Rocha, Ana Maria Bridi

**Affiliations:** 1 Departament of Animal Science, State University of Londrina (UEL), Rodovia Celso, Garcia Cid PR 445 km 380, Londrina 86057-970, Brazil; giovanna.lima.silva@uel.br (G.L.S.); apbarbon@gmail.com (A.P.A.d.C.B.); casilva@uel.br (C.A.d.S.); ambridi@uel.br (A.M.B.); 2 Departament of Preventive Veterinary Medicine, State University of Londrina (UEL), Rodovia Celso, Garcia Cid PR 445 km 380, Londrina 86057-970, Brazil; kkflaiban@uel.br; 3 CDPQ Inc., Québec, QC G1R 3X5, Canada; luiene.moura@gmail.com

**Keywords:** fatteners, slaughter, stress, physiological response, slaughtering performance

## Abstract

**Simple Summary:**

This study explores the impact of pre-slaughter stress on pigs, focusing on non-ambulatory, non-injured (NANI) pigs, and pigs showing no apparent anomalies (non-NANI). Pre-slaughter stress is known to affect animal welfare and meat quality. NANI pigs, often disabled or injured, exhibit higher body temperatures and significant blood count changes, indicating elevated stress levels. The study correlates these physiological markers with meat quality parameters, revealing that NANI pigs experience abnormalities in meat characteristics. The findings emphasize the potential use of blood biomarkers as predictive tools for evaluating pig welfare during handling, leading to improved practices and, consequently, better meat quality. This research aims to contribute to enhanced animal welfare and more efficient pork production practices.

**Abstract:**

The study aimed to investigate the physiological and meat quality differences between Non-Ambulatory, Non-Injured (NANI), and without apparent abnormalities (non-NANI) pigs in a commercial slaughterhouse setting, focusing on the impact of stress and health conditions on the overall well-being and meat quality of the animals. A total of 241 surgically castrated crossbred male pigs from Southern Brazil were analyzed, with 131 non-NANI pigs and 110 NANI pigs. Infrared orbital temperature, rectal temperature, hematological parameters, and meat quality measurements were collected. Statistical analysis included ANOVA tests and principal component analysis (PCA). NANI pigs exhibited significantly higher infrared orbital temperatures and rectal temperature (*p* < 0.01). Hematological analysis revealed higher levels of hemoglobin, hematocrit, and red blood cells in NANI pigs (*p* < 0.05). White blood cell count and lactate dehydrogenase were significantly elevated in NANI pigs (*p* < 0.01), indicating potential infections or inflammatory responses. Meat quality parameters showed that NANI pigs had lower pH values, higher luminosity, and increased drip loss (*p* < 0.01), reflecting poorer water retention and potential muscle glycogen depletion. The study highlights the physiological and meat quality differences between NANI and non-NANI pigs, emphasizing the impact of stress, health conditions, and handling procedures on the animals. Blood biomarkers proved valuable in assessing physiological stress, immune response, and potential health issues in pigs, correlating with meat quality abnormalities. Utilizing these biomarkers as predictive tools can enhance animal welfare practices and contribute to improving meat quality in the swine industry.

## 1. Introduction

Pre-slaughter handling of pigs is a highly stressful stage, impacting their systemic metabolism and bodily homeostasis [1,2,3]. Stressors during this period can be either psychological (such as social mixing, overcrowding, fights, contact with humans, and new environments) or physical (such as hunger, thirst, exhaustion, injuries, and extreme temperature changes) [4].

Stress can have a significant impact on animal welfare, leading to an increased incidence of skin lesions [5], while reducing productive efficiency due to animal deaths, carcass depreciation, and lower meat quality [1,6], depending on stress duration and intensity [1]. Incapacitated pigs at the slaughterhouse can cause quantitative losses and total or partial condemnation of carcasses [7], which are directly related to the mortality and the incidence of injured (NAI—non-ambulatory, injured) or exhausted (NANI—non-ambulatory, non-injured) pigs [8].

In modern pig production, growing concerns about animal welfare highlight the necessity to closely examine non-ambulatory pigs, categorized as either non-ambulatory, non-injured (NANI), or non-ambulatory, injured (NAI). The reported combined rates of non-ambulatory pigs exhibit a notable variability, ranging from 0.5% to 8%, underscoring significant differences across various herds and production systems. Contributing factors to this variability include divergent diagnostic criteria, diverse production practices, and potential reporting biases, reflecting concerns related to both animal welfare and economic considerations. The limited research on NANI necessitates an exploration of established risk factors associated with non-ambulatory conditions. These factors encompass gestational stressors, complications during birth, infectious diseases, environmental influences, and genetic predispositions [9,10].

High levels of acute stress in animals may serve as an indication of suboptimal welfare conditions, and have the potential to affect the meat quality of immobilized pigs [11]. The quality of meat is a multifaceted characteristic influenced by various factors, including genetics, nutrition, pre-slaughter handling, and post-mortem processing. The ultimate goal of meat quality classification is to objectively categorize carcasses based on their inherent characteristics, enabling more efficient marketing and processing strategies [12]. 

Five main categories of meat quality are widely recognized: Pale, Soft, and Exudative (PSE) Meat: PSE meat is characterized by its pale color, soft texture, and high exudative capacity. It is often associated with pre-slaughter stress-induced conditions, resulting in reduced water retention capacity and economic losses. Dark, Firm, and Dry (DFD) Meat: DFD meat exhibits a dark color, firm texture, and dry appearance. It is primarily linked to inadequate post-mortem acidification, affecting meat color, tenderness, and shelf life. Normal Red (RFN) Meat: RFN meat falls within the desirable quality range, displaying normal red color, firm texture, and adequate water retention capacity. This category represents the target for most meat producers and is associated with optimal animal welfare and processing practices. Pale, Firm, and Non-Exudative (PFN) Meat: PFN meat is characterized by its pale color, firm texture, and low exudative capacity. It is less common than PSE and DFD categories and may be associated with factors such as genetics, handling, and slaughter conditions. Red, Soft, and Exudative (RSE) Meat: RSE meat features a red color, soft texture, and high exudative capacity. It is less frequent than PSE meat and may be related to factors such as ambient temperature and post-mortem resting time [13]. 

Acute stress can lead to physiological and biochemical alterations in pigs, impacting meat quality. Among the primary changes are increased meat pH, resulting in Dark, Firm, and Dry (DFD) meat; reduced meat water-holding capacity, resulting in Pale, Soft, and Exudative (PSE) meat; and meat oxidation, leading to deterioration in color, flavor, and texture [14]. The research conducted by Carr et al. (2005) provides compelling evidence that fatigue in pigs is a contributing factor to the development of DFD (Dark and Firm, Dry—dark, firm, and dry) and PSE (Pale, Soft and Exudative—pale, soft, and exudative) anomalies in pork, leading to suboptimal meat quality [11]. The authors of this study propose that the critical phase just before slaughter induces high short-term stress, which can affect various physiological responses. One plausible effect could be an increase in metabolism, leading to an increase in body temperature. Accelerated glycolysis during antemortem and post-mortem events can result in higher lactate production and lower muscle pH. Carcasses kept at elevated temperatures show a faster rate of pH decline, possibly associated with the denaturation of membrane-bound proteins. Another conceivable result of short-term stress is the activation of metabolic systems and cascade mechanisms that persist post-mortem, contributing to a faster decline in pH and a greater likelihood of protein denaturation. 

Therefore, predicting pork quality at slaughter time is crucial from both logistical and economic perspectives, as it enables important decision making during the period between slaughter time of pigs and carcass refrigeration at slaughterhouses. These insights not only assist pig producers and meat processing companies to identifying issues related to animal welfare during pre-slaughter and slaughter practices, but they also allow for the correction of the underlying causes. Improvements of pre-slaughter handling and slaughter practices enhance not only animal welfare, but also carcass quality, preventing losses in pork production [15]. 

Physiological markers are valuable indicators to assess the stress severity in animals, allowing for the interpretation of discomfort levels, including the nature of stressors, such as hydration status, fear, muscle fatigue, and nutritional and energy status [3]. The main types of indicators are as follows: (A) causative indicators, measuring factors the cause stress; (B) biological response indicators, measuring the physiological response of organisms to cope with stress; and (C) consequence indicators, quantifying the productive consequences of physiological responses to stress [16].

Biomarkers serve as potential indicators for a variety of biological processes [17] and they can be objectively measured [18]. The ideal biomarker should demonstrate specificity for a particular condition and physiological state, ensuring safety, ease of measurement, and quantifiability [19,20]. The hypothalamus–pituitary–adrenal (HPA) axis is a vital system for regulating the stress response. The hypothalamus releases corticotropin-releasing hormone (CRH), stimulating the pituitary gland to release adrenocorticotropic hormone (ACTH). In turn, the adrenal glands release stress hormones. This activation of the HPA axis is crucial for adapting to temporary challenges, but chronic stress can lead to imbalances, negatively impacting health and contributing to various physiological problems. Maintaining a proper balance of the HPA axis is essential for overall homeostasis [21]. The HPA axis, along with the autonomic nervous system (ANS) and immune system, serves as the initial response to stress, triggering biomarkers such as cortisol, epinephrine, alpha-amylase, and proinflammatory cytokines [22]. Hormonal variations, especially cortisol and epinephrine, are recognized as classical stress markers. The baseline value, measured within minutes of exposure to stressors, differs from the basal value necessary for basic homeostasis without challenges [23,24]. Animals exhibit adaptive stress responses, involving behavioral and physiological parameters, providing valuable insights into their stress levels under psychological or physical duress [18]. 

Several studies [25,26,27,28,29,30,31,32] have evaluated physiological stress biomarkers as potential predictors of meat quality; however, the results have shown high variability which reflects the complexity of the subject. Therefore, to ensure high accuracy of the results, the present study aimed to evaluate the stress level and meat quality of NANI pigs by combining the stress biomarker analysis with the assessment of meat quality parameters.

## 2. Materials and Methods

### 2.1. Animals 

A total of 241 commercial crossbred male pigs (surgically castrated) and same genetics (synthetic cross) were analyzed at the commercial slaughterhouse located in Southern Brazil, with 131 pigs showing no apparent anomalies (non-NANI pigs) and 110 classified as a non-ambulatory, non-injured (NANI) pigs, with an average slaughter weight of 125 ± 4.2 kg. The sample size of 241 animals was not established through a formal power analysis. Instead, practical limitations, particularly the financial constraints associated with the chosen analyses, guided the selection. To balance these constraints with maintaining statistical robustness, we maximized the feasible sample size within the available budget. This approach aligns with previous statistical recommendations suggesting a minimum of 10 degrees of freedom in the error term for robust analyses, ensuring statistically meaningful results for the present study [33]. The pigs were transported from commercial farms that had the same standard feeding program, management system, similar loading facility design, and were on average 460 km from the slaughterhouse. The trucks used had three-story bodies, with a total area of 112.5 m^2^. The average journey time was eight hours, followed by a three-hour rest period after unloading. During unloading, animals that showed a combination of skin discoloration, mouth breathing, and an inability to stand up, move, and keep up with the rest of the group due to fatigue were considered to be non-ambulatory and non-injured (NANI) pigs [34] and kept in a separate stall from the other animals with no apparent abnormalities (non-NANI). These animals were identified by a slaughterhouse employee. All pigs were kept in a cage (0.6 m^2^/pig) for 3 h before slaughter, during which time they had free access to water. 

After the rest period, non-NANI pigs were handled from the holding pen to the stunning point in groups of 10–15 animals, and in the restrainer, they were managed in single file using rattle paddles and boards and occasionally electric prods when deemed necessary, for example, when pigs refuse to walk. On the other hand, the NANI pigs were sent for emergency slaughter with the assistance of a wheelbarrow. Prior to bleeding, the pigs were subjected to electronarcosis through an electric current with a voltage of 250 V applied at two points behind the ear (temporal fossae) and 120 V electrodes applied on the chest (between the 4th and 5th space intercostal to the left) with intensity of 1A and frequency of 50 Hz for 5 s. The selection of the animals occurred randomly at the time of stunning, and the chosen pigs were identified in the bleeding chute through the use of a tattoo on the left hind leg for their recognition during the experiment. To ensure standardization, the selection of the pigs was carried out consistently by a single student at the bleeding chute throughout the duration of the experiment. 

Samples from NANI and non-NANI pigs (average 10/day/group) were collected from the same batch for two weeks to ensure sample homogeneity.

### 2.2. Body Temperature 

After stunning, the carotid arteries and jugular veins were incised with an approximately 5 cm cut in the middle region of the neck (sternum bone). Immediately afterward, the rectal temperature (RT) of each animal was measured using a digital veterinary thermometer. 

The (infrared orbital temperature (IROT) was measured [35] in the left eye region using the FLIR T440^®^ thermal camera (FLIR Systems Inc., Wilsonville, OR, USA) with a 15° × 11° lens (40 mm), 640 × 480-pixel detector, thermal sensitivity < 40 mK (<0.04 °C at 30 °C ambient temperature), and temperature range from −20 °C to 350 °C. An emissivity of 0.98 was used [36]. For standardization, a distance of 1 m between the camera and the animal and an angle of 90° was adopted. The focus was manually adjusted as needed, and at least two images were captured per animal to select the one with the best resolution for further analysis.

To minimize the confounding effects of ambient temperature on IROT variation, a digital thermo-hygrometer with a data logger (HOBO ware^®^, Sigma Sensors, São José dos Campos, São Paulo, Brazil) was used to record ambient temperature (T°) and relative humidity (RH). The logger was programmed to record T° and RH at 5 min intervals during the experiment. The average ambient temperature was 22.9 °C (ranging from 16.3 to 32.2 °C) and the average of RH was 63.7% (ranging from 41.1% to 72.3%). The FLIR Tools^®^ software program (Version 6.4., Teledyne FLIR LLC, Wilsonville, OR, USA) was used to determine the maximum, minimum, and average temperatures (°C) from the obtained thermal images (Figure S1).

### 2.3. Physiological Measurements

Blood samples were collected at exsanguination in plastic cups (~150 mL) and transferred into three 4 mL vacuum tubes (FirstLab, São José do Pinhais, Brazil), of which one contained fluoride and ethylenediaminetetraacetic acid (EDTA) for lactate and glucose analysis, EDTA only for complete blood count, and while the other contained a clot activator for albumin and lactate dehydrogenase (LDH) analysis. The tubes were gently inverted eight times immediately after collection for homogenization. Tubes were transported in refrigerated containers to the laboratory where they were immediately centrifuged at 3000 rpm for 10 min at 18 °C to separate serum and plasma. Once extracted, serum and plasma samples were transferred to Eppendorf tubes and frozen at −20 °C until biochemical analyses were performed.

A veterinary automated hematology analyzer, pocH-100 iV Diff^®^ (ROCHE, São Paulo, SP, Brazil), was used to assess complete blood counts. The analyzed parameters included hemoglobin concentration (HB), hematocrit percentage (HT), red blood cells (RBC), white blood cells (WBC), segmented neutrophil percentage (Seg.), platelet count (Pl.), lymphocyte percentage (Lymph.), mean corpuscular volume (MCV), mean corpuscular hemoglobin (MCH), mean corpuscular hemoglobin concentration (MCHC), and red cell distribution width (RDW).

The detection of blood albumin, lactate dehydrogenase, lactate and glucose were measured using analytical kits (analisa^®^, LDI Flex^®^, Dimension LA^®^, Gluc Ver Flex^®^, respectively). The concentrations were performed using the Dimension^®^ Xpand Plus device (Siemens Healthcare Diagnostics Inc.—Tarrytown, NY, USA). 

### 2.4. Meat Quality Measurements

After slaughter, carcasses were eviscerated, split, and chilled according to standard commercial practices. To estimate carcass and verify the losses during the cooling period, Hot carcass weight (HCW) was recorded on the slaughter line, while cold carcass weight (CCW) was registered after overnight chilling. The percentage of weight loss during cooling (%WL) was calculated according to the equation below [37]:

%WL = 100 − (CCW × 100/HCW)

Meat quality was assessed by measuring color, pH, and drip loss at 24 h after slaughter (in the cutting room with an average cooling temperature of 4 °C). The pH was determined using a portable pH meter fitted with an integrated temperature probe (Testo 205, Testo AG, Lenzkirch, Germany), which was previously calibrated with two solutions, acidic (pH = 4) and basic (pH = 7) at room temperature. The pH measurement was taken in the left longissimus thoracis (pH LT; between the last and the second last rib) and in the left semimembranosus (pH SEM; proximal portion) muscles. The electrode was rinsed with distilled water between each measurement. At the time of measurement, the mean temperature of the longissimus thoracis was 0.9 °C, and that of the semimembranosus was 2.8 °C.

The drip loss analysis was performed according to the method described by Kauffman et al. (1986). A superficial incision was made in the overlying muscle of the proximal portion of the semimembranosus muscle to facilitate oxygenation of this region at room temperature for 10 min. To prevent desiccation of the meat sample, the region designated for analysis was enclosed with a non-contact plastic film barrier, thereby facilitating the uninhibited diffusion of oxygen before the commencement of measurements. After 10 min, the plastic was removed, and then the filter paper (# 589 Blue Ribbon, Whatman, International Co., Ltd., Mont Royal, Montreal, QC, Canada) was placed on the ham using forceps for 3 s. The filter paper was compared with the photographic standard containing the areas of wetness (score 0–5) [38]. This comparison was made by a single trained observer to avoid discrepancies in observation. The percentage of drip loss was calculated according to the equation below [38]:

% drip loss = 1.8 + 1.4 (score of the filter paper)

After completing the drip loss analysis with the filter paper, an additional 20 min were allowed for a total exposure time of 30 min to analyze the color. The portable Minolta CR-10^®^ equipment (CR-10, Konica Minolta, Inc., Osaka, Japan) was used with illuminant D65, a 10° viewing angle, and an aperture size of 8.0 mm [39] to evaluate three different points on the sample’s surface and calculate the average of these points. The results were expressed using the CIELAB system (L*, a*, b*).

Finally, the meats were classified into five pork quality categories, namely PSE (pale, soft, and exudative), PFN (pale, firm, and nonexudative), RSE (red, soft, and exudative), RFN (red, firm, and nonexudative), and DFD (dark, firm, and dry) according to pH24, light reflectance (L*), and drip loss (PG) [13] (Table 1).

### 2.5. Statistical Analysis 

Since the body temperature measurements of the pigs, hematological and biochemical parameters, and meat quality parameters satisfied the assumptions of normal distribution (Shapiro–Wilk test), the analysis of variance (ANOVA) test was used to compare these factors between NANI pigs and non-NANI pigs. The results were analyzed using jamovi software (version 2.2, 2022), and a significance level of *p* < 0.05 was set for all statistical tests.

Principal component analysis (PCA) was conducted to determine which variable best explains the data variation. The data were scaled before conducting the PCA to ensure comparability between variables with different units and scales [40]. This analysis was performed using the Python programming language.

## 3. Results and Discussion

### 3.1. Body Temperature, and Hematological and Biochemical Parameters 

NANI pigs displayed significantly higher infrared orbital temperature measurements, including maximum (IROT MAX), minimum (IROT MIN), and average (IROT AVG) values, with a *p*-value lower than 0.01 when compared to non-NANI pigs (Table 2). In addition, rectal temperature of NANI pigs was higher (*p* < 0.01) than that of non-NANI pigs, which is an important physiological indicator reflecting the health and overall condition of the animals [31]. 

NANI pigs, typically classified as disabled or injured, often present underlying health issues or injuries that can significantly affect their overall well-being. In response to illness or discomfort, pigs tend to exhibit an elevation in body temperature as part of their immune response [19]. Moreover, disabled or compromised pigs, including NANI pigs, may undergo higher levels of stress compared to their healthy counterparts [19,30]. Stress, known to elicit physiological responses in animals, can contribute to an increase in body temperature. This heightened stress level is particularly relevant during the various handling procedures that pigs experience on their way to the slaughterhouse, involving fasting, loading, transportation, unloading, housing in slaughterhouse pens, rest, and stunning [26,27,28]. These disruptions in their regular routines can be considered stressful factors, significantly impacting the welfare of the animals. Furthermore, NANI pigs may face challenges in mobility due to their disabilities or injuries, which can hinder their ability to effectively regulate body temperature [41]. Reduced mobility limits their capacity to engage in physical activities that pigs typically use to cool down [42,43,44]. 

Thus, the higher body temperatures observed in NANI pigs in this study could be attributed to a result of their compromised health conditions, stress levels, and limited mobility [42,43,44].

Values of hemoglobin (HB), hematocrit (HT), and red blood cells (RBC) were higher in NANI pigs compared to non-NANI pigs (*p* < 0.05), and both groups had HT values above the reference range for the species (HT = 5 to 8 × 10^6^/μL) [45]. Under physical stress, the spleen is compressed to release a larger number of oxygenated red blood cells and platelets (splenic contraction) to provide an increase in the physical activity of the animal [45]. Studies show that pre-slaughter procedures can result in a significant response to acute stress [29,30,31]. Therefore, in the present study, it can be assumed that NANI animals experienced higher stress levels compared to non-NANI pigs. These results may be reinforced by the difference in the mean platelet levels, where NANI pigs exhibited higher means compared to non-NANI pigs (*p* < 0.05).

No significant difference (Table 2) between NANI and non-NANI pigs were observed for the mean corpuscular volume (*p* = 0.495), the mean corpuscular hemoglobin (*p* = 0.127), the mean corpuscular hemoglobin concentration (*p* = 0.18), and red blood cell distribution width (*p* = 0.442), with all values falling within the expected range. As all results were satisfactory, it is therefore reasonable to assume that none of the animals exhibited signs of anemia [45].

NANI pigs presented a higher leukocyte count (Table 2) compared to non-NANI pigs (*p* < 0.01), and the values exceeded the normal range (leukocytosis) [45]. Inflammatory processes are characterized by an increase in white blood cells, fibrinogen concentration, total proteins, and hematocrit, as well as a reduction in hemoglobin and albumin [46]. In this study, white blood cells in NANI pigs were increased, while albumin concentration decreased compared to non-NANI pigs (*p* < 0.01), suggesting that these pigs were underwent some type of active infection, either a viral infection or an inflammation [47]. 

The results of mature neutrophils (segmented) showed a difference, with NANI pigs showing 9.15% more than non-NANI pigs (*p* < 0.01) (Table 2). On the other hand, NANI pigs presented a lymphocyte level (lymphopenia) (*p* < 0.01) below the reference value [45]. Lymphopenia, defined as a decrease in the number of circulating lymphocytes, is associated with acute infections (bacterial or viral), stress conditions, or cases of steroid therapies, where the release of glucocorticoids hinders lymphocyte production and promotes lymphocyte cell lysis [48]. The neutrophil-to-lymphocyte ratio was significantly higher in NANI pigs when compared to non-NANI pigs, indicating that NANI pigs experienced more stress than non-NANI pigs [47,49]. 

The immune system response depends on the mediator released in the process of stress: catecholamines and glucocorticoids [50]. Catecholamines, typically produced at the onset of stress or during short-term stressful stimuli, contribute to an increase in leukocytes, particularly granulocytes and lymphocytes, which are released into the systemic circulation. Conversely, cortisol, the predominant mediator in prolonged stressful situations, tends to decrease the number of lymphocytes in the bloodstream. Moreover, glucocorticoids have the capability to suppress the production of cytokines and immunoglobulins [50,51,52]. This dynamic interplay between catecholamines and glucocorticoids highlights their distinct roles in shaping the immune response during various stress conditions.

The total protein and albumin concentrations in blood are crucial indicators of protein homeostasis and both indicators usually increase with dehydration. Generally, plasma albumin concentrations follow in parallel with total protein concentrations, while dehydration is the only cause of increased albumin levels (hyperalbuminemia) [53,54]. In the present study, the albumin levels were higher in non-NANI pigs compared to NANI pigs (*p* < 0.01) and the values were considered high in relation to the reference range (1.80 to 3.30 g/dL) for both groups [53,54]. This finding suggests that dehydration was not a causal factor for NANI in the pigs in this study [47]. In the case of NANI pigs, the observation of leukocytosis (increased white blood cells) indicates the presence of an inflammatory process. However, albumin levels did not follow the expected trend of decrease. This raises the possibility that some NANI pigs may have presented subclinical infections. Subclinical infections, which do not exhibit apparent symptoms, may have induced a mild inflammatory response, leading to leukocytosis. However, the acute-phase response may have been attenuated, resulting in non-decreased albumin levels. Additional factors may have influenced albumin levels: reduced appetite, common in animals with diseases, may have contributed to the observed hypoalbuminemia [55]. 

The lactate dehydrogenase (LDH) values (Table 2) were 65% higher in NANI pigs compared to non-NANI pigs (*p* < 0.01) and both groups were above the reference range (380U/L to 634U/L), as proposed by Meyer et al. (2004) [56]. LDH is an important stress indicator enzyme to monitor the quality of pre-slaughter conditions [25,26,27,28]. Elevated levels of circulating LDH are commonly observed in instances of muscular damage, potentially linked to a greater incidence of injuries arising from factors such as loading, transportation, unloading, lairage, adverse weather conditions, and intense physical exertion [25,57,58,59]. During strenuous muscular activity and muscle damage caused by pre-slaughter handling, LDH is released into the bloodstream as a consequence of ruptures in the muscle cell membrane. As NANI pigs are typically exhausted animals, they may be more prone to developing muscular lesions compared to non-NANI pigs, leading to higher LDH values [25,60]. 

### 3.2. Meat Quality Parameters

Meat quality data presented in Table 3 shows that the final pH values were higher in non-NANI pigs (*p* < 0.01), though still within the standard range for finishing pigs [61]. Muscle pH post-mortem is an important trait which determines the quality of meat [62]. The alteration in pH is evident due to post-mortem activities in muscle tissues. The post-mortem pH of meat is influenced by factors such as lactate concentrations, the physiological condition of the muscle at stunning, and the rate of muscular energy production [63]. After slaughter, anaerobic metabolism occurs, where glycogen is converted into lactic acid, decreasing the pH of post-mortem carcass [61]. Typically, muscle pH is assessed at 1 hour (pHi) and/or 24 h (pH24) after slaughter to better describe the pH decline dynamics [64]. It is not only the extent of the pH decline but also the velocity that determine muscle protein denaturation and thus water holding capacity of the meat. In addition, ultimate pH (pH24 h) post-mortem is the best indicator of several important attributes of pork traits, including water-holding capacity, tenderness, and color [65]. In the pork loin muscle after slaughter, the pH values generally range between 6.3 and 6.7 at 1 h and between 5.5 and 6.0 at 24 h [66].

Preslaughter stress has a significant impact on meat quality, primarily influencing undesirable post-mortem pH changes, which are contingent upon the availability of muscle glycogen reserves and the metabolic activity of the muscles at the time of slaughter [67]. Prolonged preslaughter muscular activity in animals results in the depletion of muscle glycogen reserves. Insufficient muscle glycogen at the time of slaughter leads to an elevated ultimate pH of the meat, as there is an inadequate amount of glycogen to produce sufficient lactic acid and net H+ ions during post-mortem anaerobic glycolysis in the absence of blood circulation [61]. Non-NANI pigs had higher pH values than NANI pigs likely due to the depletion of their energy reserves caused by the chronic stress experienced by these animals [68]. Although it is not common for NANI pigs to have a lower pH than non-NANI pigs, a prolonged waiting period prior to slaughter can heighten animal stress, leading to an increase in cortisol levels and impacting the final pH of the meat [61]. Non-Ambulatory, Non-Injured (NANI) pigs were expedited for emergency slaughter, being prioritized as the first group for the process. Presumably due to this circumstance, NANI pigs exhibited a lower pH compared to non-NANI pigs. 

According to the results shown in Table 3, NANI pigs had lower pH levels (*p* < 0.01) in the loin and ham, and consequently higher luminosity (L*) and drip loss values compared to non-NANI pigs. This reinforces the fact that the pH plays an important role in meat quality, directly affecting coloration and water retention capacity (WRC) [69]. Meat with a lower final pH tends to have a pale color, low water retention capacity and flabby texture [70,71]. 

Low WRC leads to moisture loss and thus weight loss (WL) during carcass storage [70]. This is supported by the results of WL during cooling. NANI animals showed higher drip loss values (*p* < 0.01), thus losing more weight compared to non-NANI pigs.

**Figure 1:**
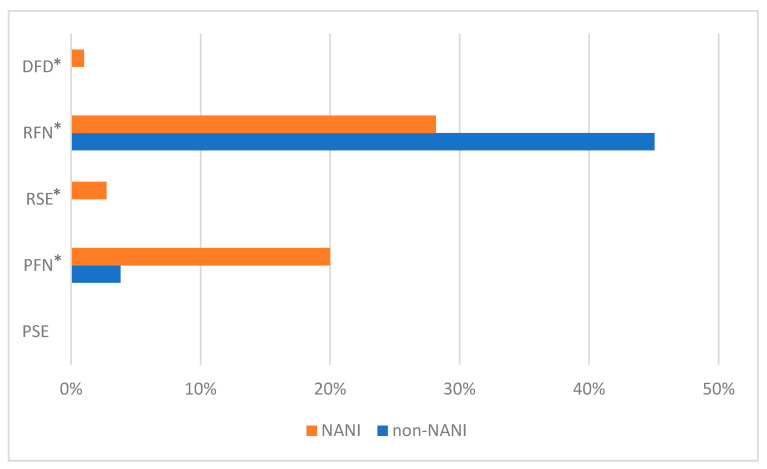
Classification of the meat quality of NANI and non-NANI pigs. NANI = non-ambulatory, non-injured; non-NANI = pigs showing no apparent anomalies. Superscript * mean that treatments are significantly different (*p* > 0.05). PSE = pale, soft, exudative; PFN = pale, firm, and nonexudative; RSE = red, soft, exudative; RFN = red, firm, and nonexudative; DFD = dark, firm, and dry.

There was a significant difference (*p* > 0.05) in the incidence of pale, firm, non-exudative (PFN) meats; reddish, soft, and exudative (RSE) meats; pink reddish, firm, and non-exudative (RFN) meats; and dark, firm, and dry (DFD) meats in both NANI and non-NANI animals (Figure 1).

The long-term, chronic stress triggers the activation of the hypothalamic–pituitary–adrenal (HPA) axis. This activation prompts the secretion of corticotrophin-releasing factor from the hypothalamus, adrenocorticotropic hormone (ACTH) from the pituitary gland, and cortisol from the adrenal cortex [28]. Cortisol release into the circulation induces catabolic activity in peripheral tissues (glycogenolysis, proteolysis, and lipolysis) and anabolic activity in the liver (gluconeogenesis and protein synthesis) to elevate blood glucose levels and provide the necessary energy to cope with the stressor [48]. Prolonged stress during the preslaughter period can rapidly intensify muscle glycogenolysis and lead to muscle glycogen depletion, resulting in reduced post-mortem lactic acid production and pork with characteristics of dark, firm, and dry (DFD) meat [72]. 

The experimental findings indicating an elevated occurrence of pink reddish, firm, and non-exudative (RFN) meats in Non-Ambulatory, Non-Injured (NANI) pigs align with the notion that potentially lower stress levels contribute to a more favorable meat quality, characterized by normal color, firm texture, and adequate water retention. Conversely, the incidence of DFD meats in NANI pigs may be attributed to accelerated glycogenesis and muscle glycogen depletion, driven by the pronounced energetic demands associated with prolonged stress [28]. The transportation of animals to slaughterhouses can be one of the possible causes of DFD meat, as it is a highly energetic activity for pigs [28].

The activation of the short-term, acute stress response triggers the release of catecholamines through the sympathetic–adrenal–medullary (SAM) axis, leading to increased blood lactate and glucose levels via rapid glycogenolysis in muscles and the liver. Consequently, acutely stressed pigs display a rapid pH drop in skeletal muscles within the first 45 min post-mortem. This, coupled with elevated meat temperature, induces denaturation of sarcoplasmic and myofibrillar proteins, reducing their water-holding capacity and resulting in pork with characteristics of PSE (pale, soft, and exudative) [73,74]. The possible explanation for the appearance of meats pale such as PFN and meats soft and exudative such as RSE is that all pigs underwent stunning shortly before the thermographic image capture. Stunning is a handling procedure that generates brief but intense stress to animals. Therefore, the body temperature of animals is elevated due to metabolic reactions in response to stress [6], which may result in more exudative meats [75].

### 3.3. Principal Component Analysis 

**Figure 2:**
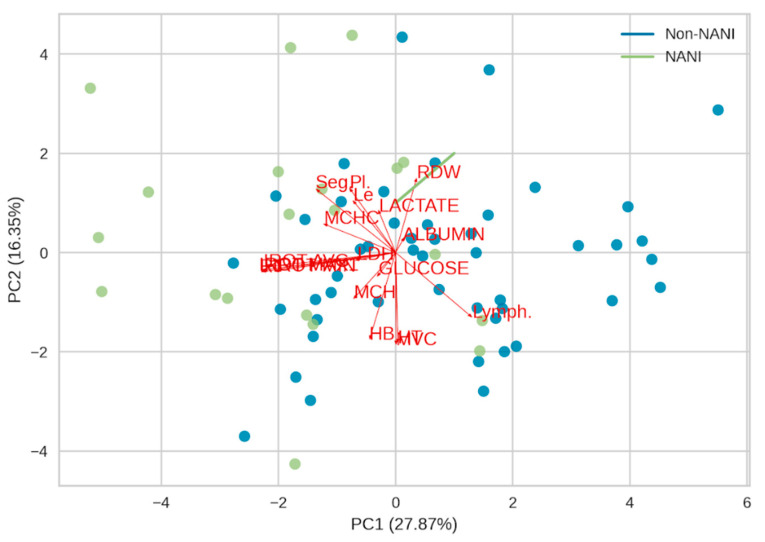
Principal component analysis with infrared orbital temperature and hematological and biochemical parameters of NANI and non-NANI pigs. PC1 = Principal Component 1; PC2 = Principal Component 2; RT = rectal temperature; HB = hemoglobin concentration; HT = hematocrit; RBC = red blood cells; MCV = mean corpuscular volume; MCH = mean corpuscular hemoglobin; MCHC = mean corpuscular hemoglobin concentration; RDW = red blood cell distribution width; WBC. = white blood cells; Seg. = segmented neutrophils; Lymph. = lymphocytes; Pl. = platelets; LDH = lactate dehydrogenase.

The principal component analysis (PCA) allowed for the characterization of the investigated variables. The PCA explained 44.22% of the total structure of variance and covariance (PC 1 (27.87%), PC 2 (16.35%)) among orbital infrared temperature, rectal temperature, hematological, and biochemical parameters in relation to NANI and non-NANI pigs (Figure 2). PC1 (39.74%): This component explains the largest portion of the variance in the data and is mainly driven by infrared orbital temperature, hemoglobin concentration, hematocrit, red blood cells, mean corpuscular volume, mean corpuscular hemoglobin, mean corpuscular hemoglobin concentration, and lactate dehydrogenase. These variables are all associated with red blood cells and oxygen transport, suggesting that this component captures differences in physiological stress between NANI and non-NANI pigs. PC2 (14.21%): This component explains a smaller portion of the variance and is mainly driven by white blood cells, segmented neutrophils, lymphocytes, and platelets. These variables are all part of the immune system, suggesting that this component captures differences in immune response between NANI and non-NANI pigs. Therefore, the PCA analysis suggests that there are two main sources of variation in the data between NANI and non-NANI pigs: Physiological stress: NANI pigs tend to have higher infrared orbital temperature, hemoglobin concentration, hematocrit, red blood cells, mean corpuscular volume, mean corpuscular hemoglobin, mean corpuscular hemoglobin concentration, and lactate dehydrogenase, all of which are associated with red blood cells and oxygen transport. This suggests that NANI pigs experience more physiological stress than non-NANI pigs [76]. 

Furthermore, the temperatures obtained using a thermal camera and rectal temperature were strongly correlated (IROT MAX *r* = 0.98, *p* < 0.001; IROT MIN *r* = 0.70, *p* < 0.001; IROT AVG *r* = 0.83, *p* < 0.001). Rectal temperature is considered a vital physiological indicator reflecting the health and physiological state of animals [76]. Under pre-slaughter stress conditions, the release of epinephrine occurs in response to the sympathetic nervous system, impacting the cardiovascular system. Increased cardiac activity induces changes in vascular tone, blood flow, and heart rate [6], leading to an elevation in body temperature [10,77]. Other researchers have provided additional support for these observations, indicating that infrared thermography can be used to predict rectal temperature [78,79]. 

Immune response: NANI pigs tend to have lower white blood cells, segmented neutrophils, lymphocytes, and platelets, all of which are part of the immune system. This suggests that NANI pigs may have a weaker immune response than non-NANI pigs [50,51,52].

The blood analysis provided information that could be used as a tool and should be associated with other signs and examinations to make a diagnosis. Pigs are more susceptible to stress compared to other mammals due to their physiology; therefore, the assessment of animal welfare needs to be conducted cautiously. The evaluation of hematological and biochemical parameters can identify changes in animal metabolism in response to stress; nevertheless, it does not replace the use of other study methods [72].

**Figure 3:**
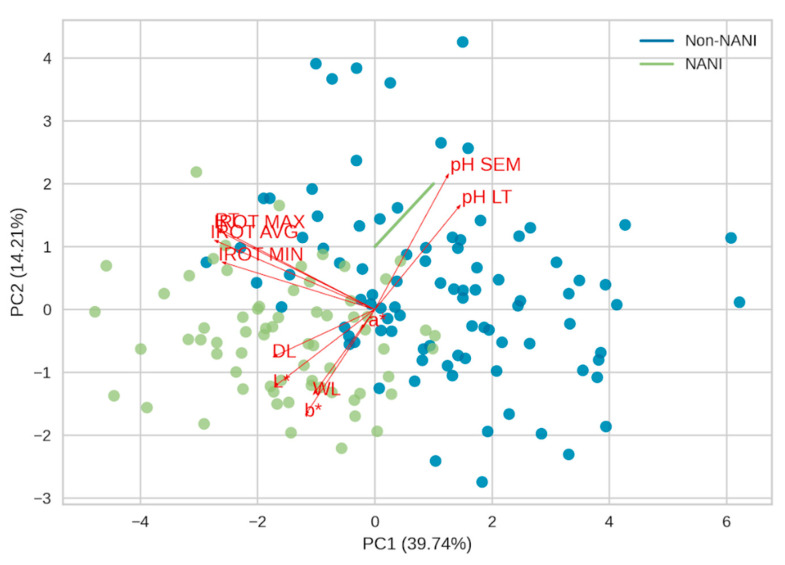
Principal component analysis with infrared orbital temperature and meat quality parameters of NANI and non-NANI pigs. PC1 = Principal Component 1; PC2 = Principal Component 2; IROT MAX = maximum infrared orbital temperature; IROT MIN = minimum infrared orbital temperature; IROT AVG = average infrared orbital temperature; RT = rectal temperature; L* = luminosity; a* = green-red component; b* = blue-yellow component; pH LT = longissimus thoracis pH; pH SEM = semimembranosus pH; WL = weight loss during cooling.

The PCA explained 53.95% of the total structure of variance and covariance for PCA 1 (39.74%) and PCA 2 (14.21%), among which includes orbital infrared temperature, rectal temperature, and meat quality parameters in relation to NANI and non-NANI pigs (Figure 3). The variables primarily located in the first quadrant of the PCA plot (infrared orbital temperature and rectal temperature) suggest a joint association, indicating that PC2 may be related to stress response (body temperature). The pH in the second quadrant and another group of variables that are in the fourth quadrant of the PCA plot (water loss, cooling weight loss, luminosity, red-green component, and blue-yellow component) appear to be highly correlated, suggesting a possible pattern in color characteristics based on water loss and on the final pH. 

Finally, the clustering of NANI animals was influenced by the temperature and final pH parameters. The exposure of animals to acute stress just before slaughter leads to the depletion of energy reserves and consequently an increase in the amount of lactic acid in the muscle, resulting in a significant pH drop in the first few hours post-mortem. Therefore, our results indicate that the condition of acute stress presented by NANI pigs can affect meat quality, favoring the occurrence of more exudative meats [9,49].

## 4. Conclusions

In our study, NANI pigs exhibited a higher body temperature and more significant changes in the blood count compared to non-NANI pigs. In an attempt to restore homeostasis, NANI pigs responded more intensely to stress and consequently, showed meat abnormalities. Therefore, the correlation between blood biomarkers and meat quality underscores the possibility of utilizing them as a predictive tool for evaluating the physiological condition of pigs during handling. This potential application can contribute to enhancing best practices in animal welfare, such as identify and treat stress or discomfort in pigs. As a result, we can improve animal welfare by adjusting handling methods, providing adequate care and creating more comfortable environments for the animals. Consequently, this leads to improvements in meat quality.

## Figures and Tables

**Table 1:** Pork quality classification including pH24, color brightness (L* value) and drip loss [13].

Quality Class ^1^	pH24	L*	Drip Loss, %
PSE	<5.5	>50	>5
PFN	5.5 to 5.8	>50	<5
RSE	5.6 to 5.8	42 to 50	>5
RFN	5.6 to 5.8	42 to 50	<5
DFD	>6.1	≤42	<2

^1^ PSE = pale, soft, exudative; PFN = pale, firm, nonexudative; RSE = red, soft, exudative; RFN = red, firm, nonexudative; and DFD = dark, firm, dry.

**Table 2:** Analysis of variance (mean ± standard error) of infrared orbital temperature, rectal temperature, and hematological and biochemical parameters of NANI and non-NANI pigs.

Parameters ^1^	Non-NANI	NANI	*p*
IROT MAX (°C)	34.83 ± 0.12	35.92 ± 0.1	<0.01
IROT MIN (°C)	28.43 ± 0.16	30.12 ± 0.1	<0.01
IROT AVG (°C)	31.76 ± 0.12	32.84 ± 0.09	<0.01
RT (°C)	38.68 ± 0.07	39.27 ± 0.05	<0.01
HB (g/dL)	14 ± 0.13	15.07 ± 1.99	0.03
HT (%)	42.36 ± 0.47	46.83 ± 1.6	0.014
RBC (×10^6^/μL)	8.33 ± 0.09	9.32 ± 0.23	0.003
MCV (fL)	50.68 ± 0.42	49.56 ± 1.54	0.495
MCH (pg)	16.68 ± 0.19	17.43 ± 0.43	0.127
MCHC (%)	33.3 ± 0.15	32.53 ± 0.52	0.18
RDW (%)	21.79 ± 4.17	18.52 ± 0.63	0.442
WBC (/μL)	17,160.52 ± 589.48	20,498.12 ± 2478.32	<0.01
Seg. (%)	10,549.67 ± 489.45	11,612.18 ± 1923.24	<0.01
Lymph. (%)	8499.61 ± 494.315	7483.86 ± 1242.59	<0.01
Pl. (/mm)	37,298.9 ± 16,875.51	183,804.92 ± 50,678.34	0.01
Albumin (g/dL)	4.67 ± 0.05	4.42 ± 0.07	0.005
Glucose (mg/dL)	90.59 ± 1.98	87.20 ± 2.06	0.237
Lactate (mmol/L)	13.87 ± 0.52	15.06 ± 0.62	0.144
LDH (U/L)	1348.31 ± 272.27	6516.28 ± 1364.37	<0.01

^1^ IROT MAX = maximum infrared orbital temperature; IROT MIN = minimum infrared orbital temperature; IROT AVG = average infrared orbital temperature; RT = rectal temperature; HB = hemoglobin concentration; HT = hematocrit; RBC = red blood cells; MCV = mean corpuscular volume; MCH = mean corpuscular hemoglobin; MCHC = mean corpuscular hemoglobin concentration; RDW = red cell distribution width; WBC = white blood cells; Seg. = segmented neutrophils; Lymph. = lymphocytes; Pl. = platelets; LDH = lactate dehydrogenase.

**Table 3:** Analysis of variance (mean ± standard error) of meat quality parameters in NANI and non-NANI pigs.

Parameters ^1^	Non-NANI	NANI	*p*
pH LT ^[1]^	5.84 ± 0.01	5.78 ± 0.01	<0.01
pH SM ^[2]^	6.04 ± 0.02	5.95 ± 0.01	<0.01
L* ^[2]^	46.85 ± 0.1	49.49 ± 0.14	<0.01
a* ^[2]^	3.39 ± 0.09	3.46 ± 0.09	0.551
b* ^[2]^	11.95 ± 0.08	12.92 ± 0.12	<0.01
Drip Loss (%) ^[2]^	1.86 ± 0.03	3.52 ± 0.13	<0.01
WL (%)	2.1 ± 0.02	2.24 ± 0.02	<0.01

^1^ pH LT = pH longissimus thoracis after 24 h; pH SM = pH semimembranosus after 24 h; L* = luminosity; a* = green-red component; b* = Blue-yellow component; WL = weight loss during cooling; ^[1]^ Measured in the Longissimus thoracis muscle; ^[2]^ Measured in the Semimembranosus muscle.

## Data Availability

The data obtained from the slaughterhouse for this study are not openly available due to privacy concerns. However, they can be made available upon request from the corresponding author. Please contact the corresponding author for access to the data.

## Supplementary Materials

The following supporting information can be downloaded at: https://www.mdpi.com/article/10.3390/ani14050700/s1, Figure S1: Anatomical sites of orbital region (infrared orbital temperature—IROT) used for the infrared thermography assessment.
