# Supplementary material for: Animal Welfare Assessment and Meat Quality through Assessment of Stress Biomarkers in Fattening Pigs with and without Visible Damage during Slaughter
Source: Animals (Basel). 2024 Feb 23;14(5):700. doi: 10.3390/ani14050700 (PMC10931360; doi:10.3390/ani14050700)
Supplement: Supplementary file 1 [file animals-14-00700-s001.zip › animals-2850270-supplementary.pdf]

animals

MDPI

Supplementary Materials

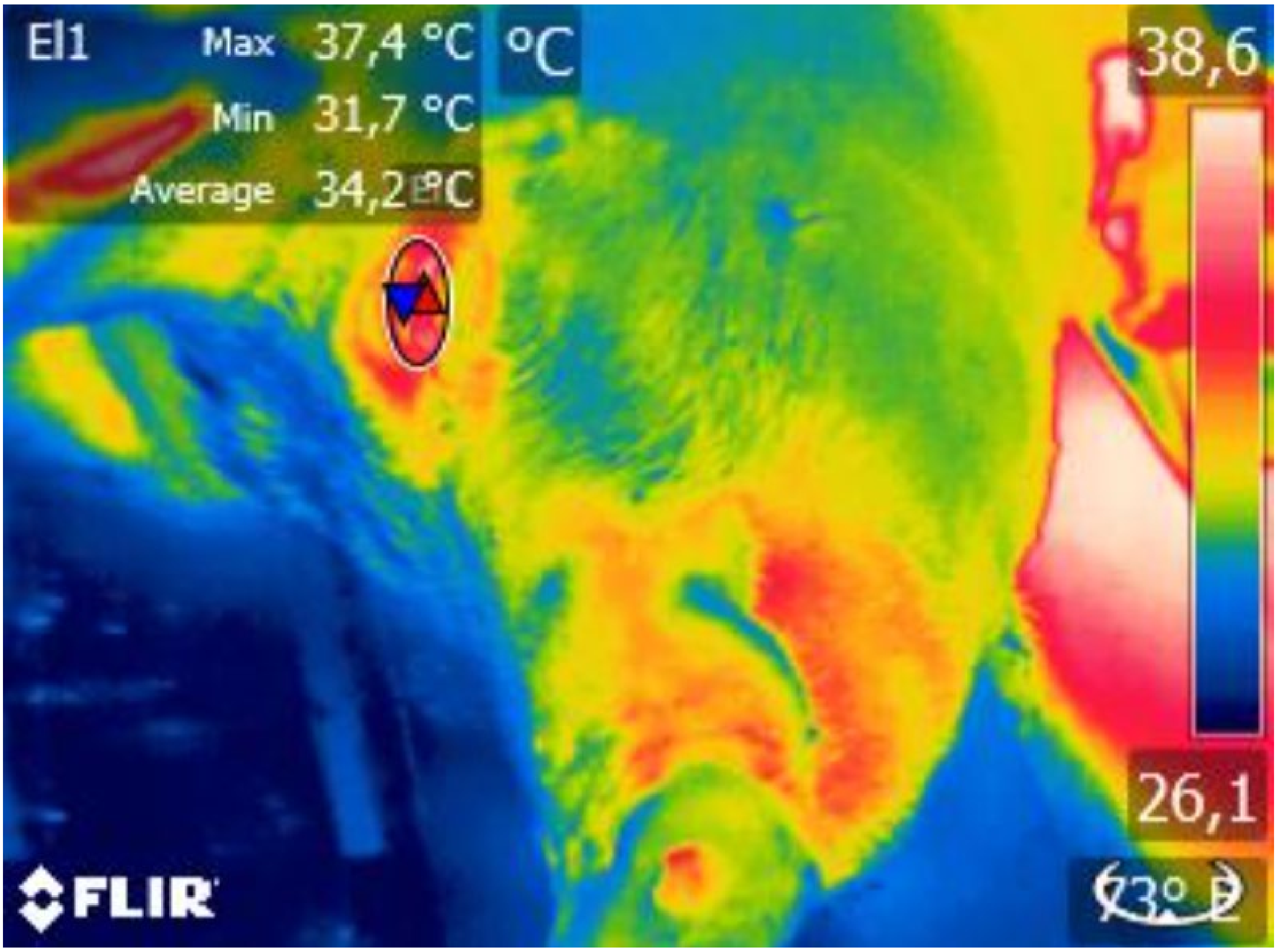

Figure S1. Anatomical sites of orbital region (infrared orbital temperature—IROT) used for the infrared thermography assessment.
